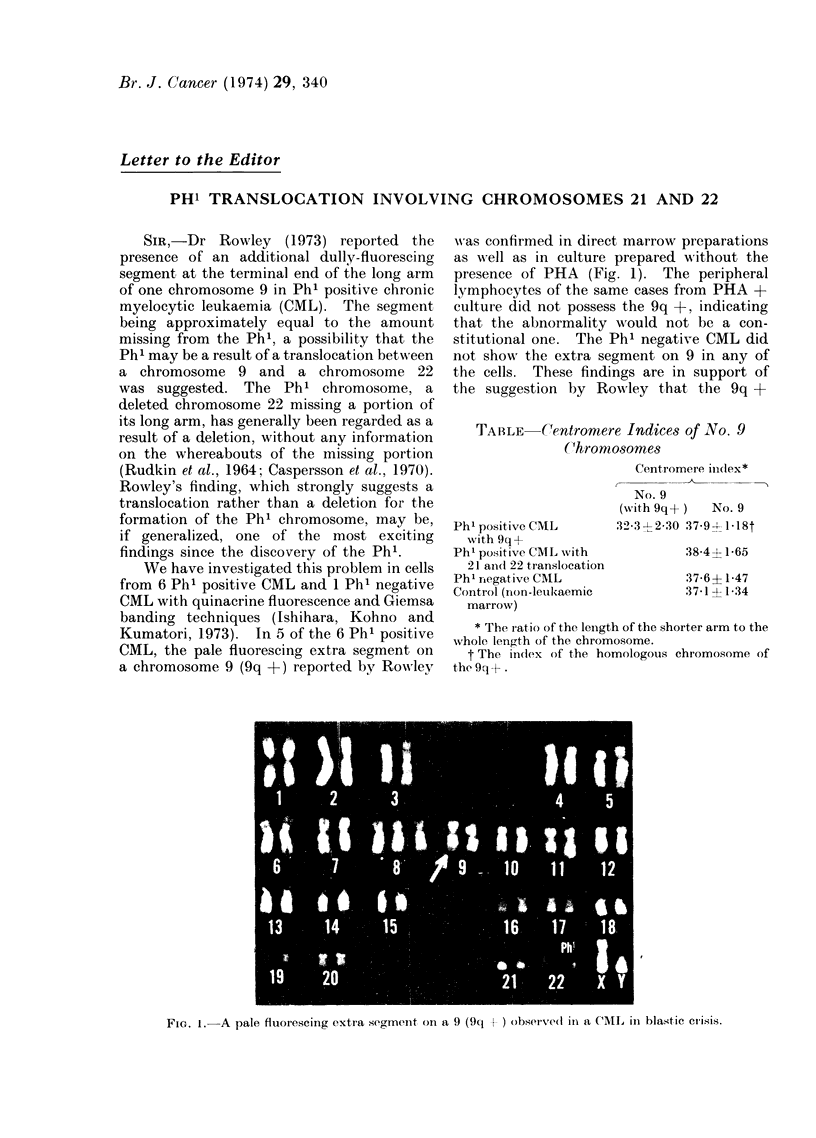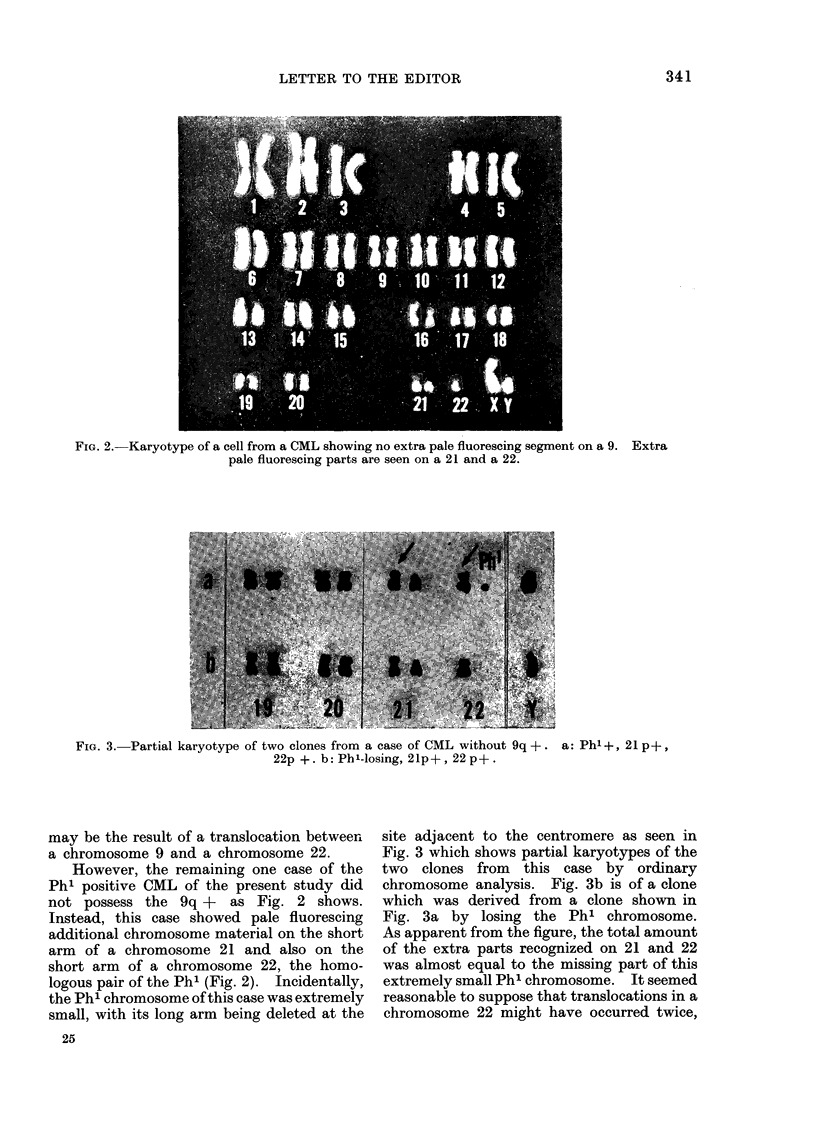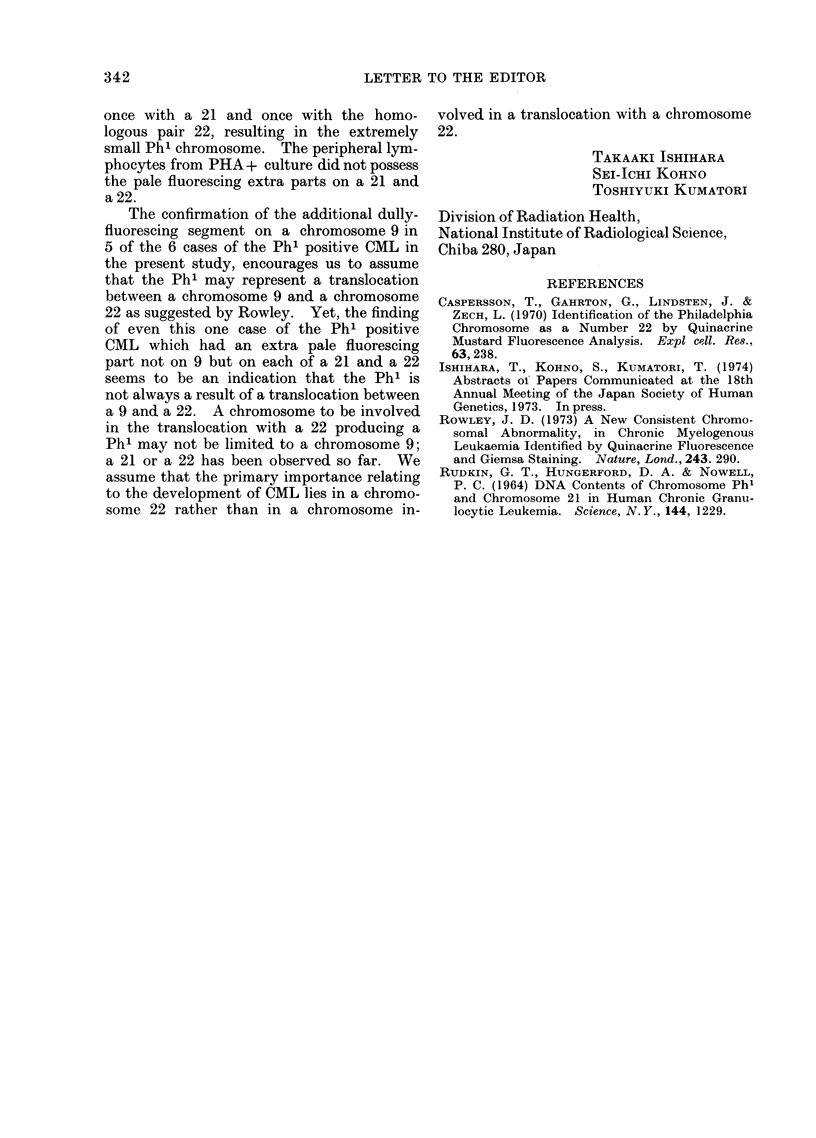# Letter: PH1 translocation involving chromosomes 21 and 22.

**DOI:** 10.1038/bjc.1974.77

**Published:** 1974-04

**Authors:** T. Ishihara, S. Kohno, T. Kumatori

## Abstract

**Images:**


*Br. J. Cancer* (1974) **29**, 340

### *Letter to the Editor*

### PH[1] TRANSLOCATION INVOLVING CHROMOSOMES 21 AND 22

SIR,—Dr Rowley (1973) reported the presence of an additional dully-fluorescing segment at the terminal end of the long arm of one chromosome 9 in Ph[1] positive chronic myelocytic leukaemia (CML). The segment being approximately equal to the amount missing from the Ph[1], a possibility that the Ph[1] may be a result of a translocation between a chromosome 9 and a chromosome 22 was suggested. The Ph[1] chromosome, a deleted chromosome 22 missing a portion of its long arm, has generally been regarded as a result of a deletion, without any information on the whereabouts of the missing portion (Rudkin *et al.*, 1964; Caspersson *et al.*, 1970). Rowley's finding, which strongly suggests a translocation rather than a deletion for the formation of the Ph[1] chromosome, may be, if generalized, one of the most exciting findings since the discovery of the Ph[1].

We have investigated this problem in cells from 6 Ph[1] positive CML and 1 Ph[1] negative CML with quinacrine fluorescence and Giemsa banding techniques (Ishihara, Kohno and Kumatori, 1973). In 5 of the 6 Ph[1] positive CML, the pale fluorescing extra segment on a chromosome 9 (9q +) reported by Rowley was confirmed in direct marrow preparations as well as in culture prepared without the presence of PHA (Fig. 1). The peripheral lymphocytes of the same cases from PHA + culture did not possess the 9q +, indicating that the abnormality would not be a constitutional one. The Ph[1] negative CML did not show the extra segment on 9 in any of the cells. These findings are in support of the suggestion by Rowley that the 9q +

TABLE—*Centromere Indices of No. 9 Chromosomes*

| | Centromere index* | |
|---|---|---|
| | No. 9 (with 9q+) | No. 9 |
| Ph[1] positive CML with 9q+ | 32·3 ± 2·30 | 37·9 ± 1·18† |
| Ph[1] positive CML with 21 and 22 translocation | | 38·4 ± 1·65 |
| Ph[1] negative CML | | 37·6 ± 1·47 |
| Control (non-leukaemic marrow) | | 37·1 ± 1·34 |

* The ratio of the length of the shorter arm to the whole length of the chromosome.

† The index of the homologous chromosome of the 9q+.

FIG. 1.—A pale fluorescing extra segment on a 9 (9q + ) observed in a CML in blastic crisis.

FIG. 2.—Karyotype of a cell from a CML showing no extra pale fluorescing segment on a 9. Extra pale fluorescing parts are seen on a 21 and a 22.

FIG. 3.—Partial karyotype of two clones from a case of CML without 9q +. a: Ph$^1$+, 21 p+, 22p +. b: Ph$^1$-losing, 21p+, 22 p+.

may be the result of a translocation between a chromosome 9 and a chromosome 22.

However, the remaining one case of the Ph$^1$ positive CML of the present study did not possess the 9q + as Fig. 2 shows. Instead, this case showed pale fluorescing additional chromosome material on the short arm of a chromosome 21 and also on the short arm of a chromosome 22, the homologous pair of the Ph$^1$ (Fig. 2). Incidentally, the Ph$^1$ chromosome of this case was extremely small, with its long arm being deleted at the site adjacent to the centromere as seen in Fig. 3 which shows partial karyotypes of the two clones from this case by ordinary chromosome analysis. Fig. 3b is of a clone which was derived from a clone shown in Fig. 3a by losing the Ph$^1$ chromosome. As apparent from the figure, the total amount of the extra parts recognized on 21 and 22 was almost equal to the missing part of this extremely small Ph$^1$ chromosome. It seemed reasonable to suppose that translocations in a chromosome 22 might have occurred twice, once with a 21 and once with the homologous pair 22, resulting in the extremely small Ph[1] chromosome. The peripheral lymphocytes from PHA+ culture did not possess the pale fluorescing extra parts on a 21 and a 22.

The confirmation of the additional dully-fluorescing segment on a chromosome 9 in 5 of the 6 cases of the Ph[1] positive CML in the present study, encourages us to assume that the Ph[1] may represent a translocation between a chromosome 9 and a chromosome 22 as suggested by Rowley. Yet, the finding of even this one case of the Ph[1] positive CML which had an extra pale fluorescing part not on 9 but on each of a 21 and a 22 seems to be an indication that the Ph[1] is not always a result of a translocation between a 9 and a 22. A chromosome to be involved in the translocation with a 22 producing a Ph[1] may not be limited to a chromosome 9; a 21 or a 22 has been observed so far. We assume that the primary importance relating to the development of CML lies in a chromosome 22 rather than in a chromosome involved in a translocation with a chromosome 22.

TAKAAKI ISHIHARA
SEI-ICHI KOHNO
TOSHIYUKI KUMATORI

Division of Radiation Health,
National Institute of Radiological Science,
Chiba 280, Japan

REFERENCES

CASPERSSON, T., GAHRTON, G., LINDSTEN, J. & ZECH, L. (1970) Identification of the Philadelphia Chromosome as a Number 22 by Quinacrine Mustard Fluorescence Analysis. *Expl cell. Res.*, **63**, 238.

ISHIHARA, T., KOHNO, S., KUMATORI, T. (1974) Abstracts of Papers Communicated at the 18th Annual Meeting of the Japan Society of Human Genetics, 1973. In press.

ROWLEY, J. D. (1973) A New Consistent Chromosomal Abnormality, in Chronic Myelogenous Leukaemia Identified by Quinacrine Fluorescence and Giemsa Staining. *Nature, Lond.*, **243**. 290.

RUDKIN, G. T., HUNGERFORD, D. A. & NOWELL, P. C. (1964) DNA Contents of Chromosome Ph[1] and Chromosome 21 in Human Chronic Granulocytic Leukemia. *Science, N.Y.*, **144**, 1229.